# Silver Nanoparticles for Waste Water Management

**DOI:** 10.3390/molecules28083520

**Published:** 2023-04-17

**Authors:** Geetha Palani, Herri Trilaksana, R. Merlyn Sujatha, Karthik Kannan, Sundarakannan Rajendran, Kinga Korniejenko, Marek Nykiel, Marimuthu Uthayakumar

**Affiliations:** 1 Institute of Agricultural Engineering, Saveetha School of Engineering, Saveetha Institute of Medical and Technical Sciences, Chennai 602105, India; kesangee@gmail.com; 2 Department of Physics, Faculty of Science and Technology, Airlangga University, Surabaya 60115, Indonesia; 3 Department of Biomedical Engineering, JNN Institute of Engineering, Kannigaipair 601102, India; merlynsujatha@gmail.com; 4 Chemical Sciences Department and the Radical Research Centre, Ariel University, Ariel 40700, Israel; karthikkannanphotochem@gmail.com; 5 Faculty of Material Engineering and Physics, Cracow University of Technology, al. Jana Pawła II 37, 31-864 Kraków, Poland; kinga.korniejenko@pk.edu.pl (K.K.); marek.nykiel@pk.edu.pl (M.N.); 6 Department of Mechanical Engineering, Kalasalingam Academy of Research and Education, Krishnankoil 626126, India; m.uthayakumar@klu.ac.in

**Keywords:** dye removal, agriculture wastes, water management, silver nanoparticle

## Abstract

Rapidly increasing industrialisation has human needs, but the consequences have added to the environmental harm. The pollution caused by several industries, including the dye industries, generates a large volume of wastewater containing dyes and hazardous chemicals that drains industrial effluents. The growing demand for readily available water, as well as the problem of polluted organic waste in reservoirs and streams, is a critical challenge for proper and sustainable development. Remediation has resulted in the need for an appropriate alternative to clear up the implications. Nanotechnology is an efficient and effective path to improve wastewater treatment/remediation. The effective surface properties and chemical activity of nanoparticles give them a better chance to remove or degrade the dye material from wastewater treatment. AgNPs (silver nanoparticles) are an efficient nanoparticle for the treatment of dye effluent that have been explored in many studies. The antimicrobial activity of AgNPs against several pathogens is well-recognised in the health and agriculture sectors. This review article summarises the applications of nanosilver-based particles in the dye removal/degradation process, effective water management strategies, and the field of agriculture.

## 1. Introduction

Rapid progress has made nanotechnology research integral to metal nanoparticles in recent years. Nanoparticles are 1–100 nm in diameter and have distinctive properties (physicochemical). Nanoparticles can be described by their massive surface-to-volume ratios in quantum effects and electronic attributes [1,2]. The rapid growth of this nanotechnology brings new options in several fields: agriculture, pharmaceutical, engineering, text, etc. [3,4,5]. Noble-metal nanomaterials with, among others, a bottom-up approach with discrete morphologies such as cubes, spheres, wires, flowers, and stars; synthetic versatility; and low cost show notable chemical/physical properties that allow distinctive interactions with the environment [6,7,8,9]. Especially purely made inorganic nanoparticles, such as gold, silver, copper, etc., have distinctive photothermal and optical properties and absorb of a broad area of electromagnetic radiation (visible spectrum). Nanoparticles of gold (Au) and silver (Ag) have magnified the properties of optoelectronic biocompatibility and stability [10]. Silver nanoparticles (AgNPs) have great market value and hold good antibacterial properties.

**Figure 1:**
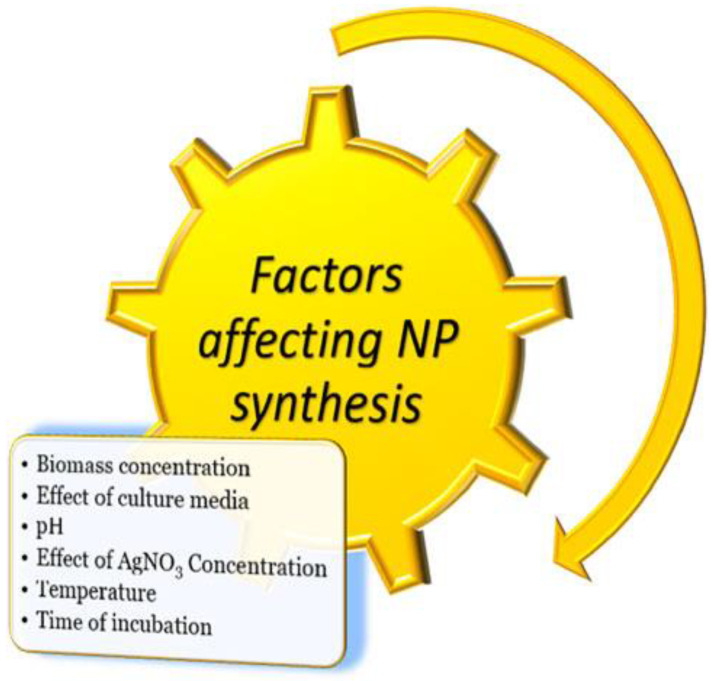
Factors that have an effect on the synthesis of metallic nanoparticles (adapted from [21]).

**Figure 2:**
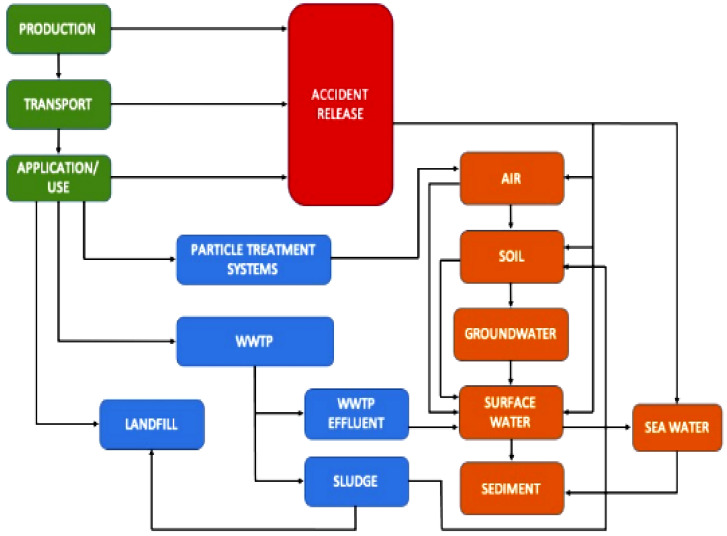
AgNPs’ release pathways and associated impacts on the environment (reprinted with permission from [14]).

Therefore, they have been used in different fields and products for industrial purposes, including therapeutics, biomaterials, sensing, food, dye-sensitised solar cells, catalysis, and photocatalysis, and the effect of the factors on synthesis is shown in Figure 1. Their production is expected to reach roughly 800 tones in 2025 [11]. The successful utilisation of silver nanoparticles (AgNPs) against water pollutants (heavy metals and organic materials) as plasmonic sensors works for photocatalysts that encourage the degradation (oxidation) of pesticides and dyes, amplifying the environmental functions [7,8,12]. Today, engineered nanomaterials are receiving more attention to study their environmental impact. Many works have been devoted to surviving the toxicity of silver nanoparticles in flowing environments [13,14]. The toxicity mechanism is still the subject of argument, but the main toxicity factors depend on the size and coating surface [15,16]. According to the literature, the toxicity of silver nanoparticles (AgNPs) is partly associated with their liberation and dissolution of Ag+ ions, although it is difficult to evaluate the relative contribution of AgNPs to this development. The toxicity of silver nanoparticle levels differs conditionally on the accumulation of exposure, and their highest levels vary in every taxon and depend on the biological community that appears in the environment [17,18,19,20]. The arrival of AgNPs in various environmental compartments as a result of their release is shown in Figure 2 (production/application use, green colour; the environmental compartments, brown colour; and technical compartments, blue colour).

The term nano-remediation currently refers to the application of nanotechnology that contains the use of fabricated nanomaterials to clean contaminated phases such as air, soil and water, groundwater, and polluted/wastewater [22,23]. Well-established technology holds potential and efficacy, but full-scale applications have some drawbacks to overcome. Engineered nanomaterials (ENMs) have many strange features such as size, shape, energy surface, and chemical core, showing sustainability that affects their end properties, and seawater reflects their released complex properties [11].

Nanoparticles have remarkable potential for use in environmental scanning applications, and silver nanoparticles exclusively have versatile, very simple-to-prepare, and low-cost materials [24,25]. Nanoparticles have been used in agricultural output, especially in crop growth and yield, in a variety of ways. Among several nanoparticles in metals, the silver nano-particle in particular is gaining a lot of attention in the fields of crop production, enhancement, detection of plant disease, and pest management. The nanoparticles of Ag, TiO_2_, Fe, Cu, Zn, Mo, Mn, Zn, carbon nanotubes, and several nanometals are used for plant pesticides and fertilisers. AgNPs have been reported to be used for sustainability, crop yield, crop improvement, and pest control. Antibacterial and fungal activities protect the crop and improve the regulation of plant nutrition [26]. Ultimately, deserved innovations in the applications of nanomaterials in the broad area of agriculture would be an impressive development in the future. They transform agricultural procedures and food production into the proper sustainable agricultural products.

The methodology of the research provided on the systematic review was provided using the following databases: ScienceDirect, Scopus, ACS Publications, Wiley Online Library, IEEE Xplore Digital Library, and Google Scholar. Keywords were associated with silver nanoparticles and water treatment, especially dye removal. This review summarises the potential solutions in dye removal treatment by silver nanoparticles (AgNPs) in the examination, remediation, and applications of water systems in agriculture, with a specific importance on their environmental safety challenges, especially taking into account the articles from the last 5 years. Several articles describe the recent progress on the review topic and perspectives of the development of technology based on silver nanoparticles.

## 2. AgNPs in Wastewater Management

The metallic AgNPs have excellent beneficial properties for a wide range of sectors utilized other than wastewater treatment, such as biology, coating, DNA sequencing, food products, drug therapy, cosmetics, biomedicine, and other varieties have been covered [27,28]. However, much of the AgNP research focuses on the antimicrobial activity against the several types of microorganisms and is related to water purification, dye removal, and wastewater treatments [29]. The synthesis of AgNPs can be based on reproducibility and a cost-effective manner, and synthesis methods depend on the differences in reactants and reaction conditions during the process [30]. Green synthesis of AgNPs from either plants or microorganisms has been surrounded by intra and extracellular approaches. Extracellular methods have commonly been preferred to avoid the difficulty of extracting intracellular AgNPs for down-streaming processing. Biological methods are more environmentally friendly and cost-effective than physical/chemically synthesised methods [31,32]. AgNP characterisation has been used to analyse the properties, but the most basic characterisation method for qualitative analysis is to show the visualisation of colour changes [33]. The degradation of toxic chemicals in aqueous solution using AgNPs has occurred in two ways: first, commonly used AgNPs assist in reducing the contaminants using chemical reducing agents by catalytic reduction. Moreover, AgNPs are used under the induced light degradation method called catalytic degradation [27].

## 3. Effects of Nanoparticle Composites in Textile Dye Removal

The sizes range from 1 to 100 nm in different distinctive features that are not found in their bulk configurations. The chemical reactivity of nanoparticles (NPs) in all fields is attributed to the significance of high available surface area. The new advancement in this regard makes use of combined membranes with biogenic nanoparticles to degrade toxic chlorinated mixtures. Functional classes of nanoparticles such as carbonaceous, zeolite, dendrimers, and metal-containing nanoparticles are used in the process of waste purification [34]. Dendrimer ultrafilters apply more strong working pressures in high-molecular-mass solutes in the range between 1000 and 3000 Da than micro- and nanofillers. Metal-containing nanoparticles play the antibacterial activity against gram-positive bacteria and have a negative and efficient method to kill the number of bacteria and biocides [35]. In addition, heavy metals are easily removed from arsenic and halogens. Zeolite removes heavy metals from water as an ion-exchange medium [36]. Carbonaceous substances can act as sorbents in an aqueous environment in organic solutes. Experimental studies have discussed better-performing enzyme reactions that perform better in biologically synthesised functionalized nanoparticles with a membrane than enhanced nanoparticle stability with single-phase reaction [34]. Table 1 explains the tabulated nanoparticles for textile dye removal. The fabricated nanoparticles were prepared by conventional methods as well as from several textile dyes with ranges from 65 to 99% through catalytic and photocatalytic degradation processes. Additionally, novel degradation processes, such as enzymatic and biogenic processes, showed 80–95% textile dye degradation [37]. The novel combination degradation method, involving photocatalytic and microwave-assisted methods, showed better removal efficiency (85%) for textile dye [38]. Parametrically optimised synthesis and adsorptive performance for the magnetic nanocomposite of chitosan-benzil/ZnO/Fe_3_O_4_ showed the best removal recorded in 98.8% of Remazol Brilliant Blue R dye (RBBR). The adsorptive mechanism in this nanocomposite explained the multi-interactions that are electrostatic attractions, hydrogen and H bonding, and interactions of n–π and π–π. This nanocomposite is suggested to be a promising composite in biosorption for the removal of anionic dyes from an aqueous environment [39].

A Schiff base cross-linked hybrid inorganic–organic synthesised nanocomposite (CS-GLA/TNC) showed an effective bio-absorbent and improved the removability of reactive azo dyes (RR120 dye) from an aqueous environment. It achieved the highest adsorption capacity recorded at 103.1 mg/g and involved the mechanism of many interactions (electrostatic attraction, n–π stacking, and H bonding) [49]. The magnetic Schiff base nanocomposite of CHT-GLA/ZnO/Fe_3_O_4_ (chitosan-glutaraldehyde/zincoxide/Fe_3_O_4_) was fabricated to remove the dye in Remazol Brilliant Blue R through an effective mechanism of adsorption. The Box–Behnken design-based optimisation method was employed for the fabrication of the magnetic adsorbent against dye degradation. This study showed that the highest RBBR-removal efficiency (75.8%) was achieved *using* the multi-interaction mechanism [52,61]. Alcantara-Cobos et al. [62] studied the coupled process of adsorption and photocatalytic degradation (adsorption–photocatalysis). The tartrazine removal study explained the preparation of ZnO nanoparticles and zeolite-ZnO composites for a coupled (adsorption–photocatalysis) process. The ZnO nanoparticles (nanoZnO) showed better efficiency compared to the composite in the processes of adsorption and degradation inclusive of UV light. Furthermore, nanoZnO was difficult to *remove* from the aqueous solution [49].

During the degradation of the photocatalytic process in methylene blue, methyl violet (cationic) and acid violet (anionic) dyes were synthesised by synthesised TiO_2_ doped on microcellulose nanocomposite (TiO_2_ + MC). This study showed that the combination of photocatalytic degradation of TiO_2_ + MC + H_2_O_2_ with the hydrogen peroxide-assisted process removed 200 mg/L (99%) of methylene blue (MB) in 150 min, and 6–7 h were required to complete the removal of the methyl violet and acid violet dyes. The mechanism of dye degradation is combined with adsorption and direct photocatalytic oxidation (by hydroxyl radicals (OH)) by nanocomposite (TiO_2_ + MC). The integrated process of AOPs (advanced oxidised process) followed by adsorption, biological treatment, and sand filtration is widely used for complete industrial wastewater [62]. The nanocomposite of single molecular pectin-starch magnetite hybrid nanoparticles showed higher efficiency of removing methylene blue dye from an aqueous solution. This adsorption depends on temperature and pH, and the hybrid decomposes magnetite temperatures between 250 and 550 °C. The developed nanocomposite showed higher adsorption efficiency and additional benefits such as lower polymer concentration, ease of synthesis, cost-effectiveness, environmental friendliness, and the absence of secondary pollutants [63].

**Figure 3:**
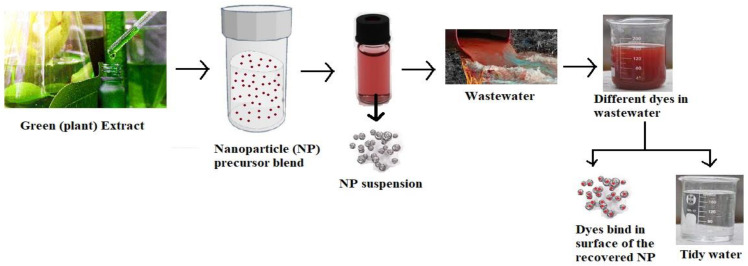
Hypothesis behind the synthesis of nanoparticle and dye remediation (adapted with permission from [55]).

Physical, chemical, and biological methods are receiving less attention due to their high costs, low efficiency, and low biodegradability. Rashid et al. [64] explained that the advanced oxidation process (AOP) is another method of removing/degrading dyes from industrial effluents [64]. Figure 3 shows the general hypothesis behind the removal of nanoparticles and dyes. The AOP discussed in determining the dye degradation/removal of organic contaminants of the dyes is oxidized by highly reactive species, which are OH (hydroxy radicals), H_2_O_2_ (hydrogen peroxide), SO_4_ (sulfate radical), and O_3_ (ozone). The above-mentioned process (AOP) provides satisfactory or potential degradation of dyes from industrial effluents and other contaminants, unlike another conventional process [3]. The fabrication of a Ni nanoparticle coated with filter paper (Ni@FP material) showed strong magnetic ability and strong antibacterial activity, explaining that an optimum photocatalytic degradation reached 93.4%. This study showed a low-cost material composite (Ni@FP) [65].

## 4. Silver Nanoparticles-Composite Activity for Wastewater Treatment

The role of the noble material silver has been studied and used in different fields of applications focused mainly on medicine and water treatment. Now, silver has rebuilt its character and performance in various forms as a nanoparticle. The biological/green synthesis of AgNPs reforms and maintains a safe environment from harmful works created by the enormous utilisation of chemicals (organic/inorganic) and *addition of* metal salt. Furthermore, the silver NP supplies are free from the use of stabilizing agents in the *manufacturing* system for chemical and physical processes [27,36]. Several research studies have discussed that the fabrication of silver nanoparticles (NPs) from various natural/biological fields and their application in the effluent/wastewater removed dyes.

Silver nanoparticles (AgNPs) have been used to remove organic pollutants/dyes from wastewater and are presented in Table 2. The fabricated hybrid aerogel graphene–carbon sphere decorated with AgNPs (G/AgCS) used the reduction of anionicdye (CR/congo red) and cationic dye (MB/methylene blue) in the presence of NaBH_4_. Furthermore, hydrogels supported by the prepared reduced graphene oxide in polyethyleneimine (PEI) have been utilised to examine the degradation (photocatalytic) of methylene blue and rhodamine B solutions [66,67,68]. Silver NPs are capable of being used for the fast destruction/degradation of organic pollutants reduced into toxic/harmful materials [27]. Induced biogenic AgNPs extracted from Citrus paradisi degrade and speed up the reduction rate of toxic chemicals in the textile industry wastewater [69]. The fabricated silver nanocomposites (Ag@MGO-TA/Fe^3+^) showed excellent performance of catalytic reduction and antimicrobial activity [70]. The piezoelectric thin film (FTO/BaTiO_3_/AgNPs) produced by the tape-casting method with the deposition of barium titanate/AgNPs degraded the pollutants of methylene blue and ciprofloxacin (pharmaceutical) in wastewater using piezo-photocatalytic degradation. The AgNPs and nanocomposites described above show great potential for several environmental applications with functional implications.

**Figure 4:**
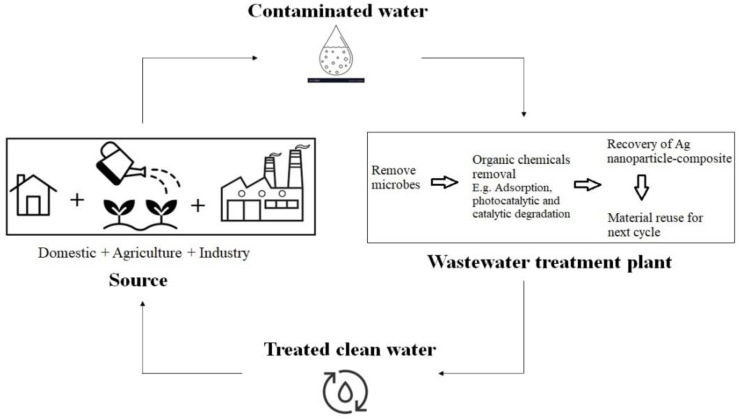
Flowchart for the silver nanoparticle compound for wastewater treatment (adapted from [27]).

Figure 4 shows the flowchart of silver nanoparticle–composite-treated wastewater for various industries. Metal nanoparticle-based nanocomposites with graphene oxide (GO) have acquired a wide range of potential applications in a number of material science fields. An efficient photocatalyst supported on nanocomposite (GO/ZnO) with metal nanoparticles was synthesised by the one-pot method. The synthesized GO–ZnO–Ag nanocomposite achieved 100% MB dye removal at 40 min of sunlight irradiation. Thus, the silver-based nanocomposite shows potential to be an effective photocatalyst for organic dyes in industrial effluents/wastewater [68].

The dye removal mechanism using AgNPs includes the adsorption onto AgNPs combined with loaded activated carbon or degradation through catalytic/photocatalytic methods or in combination with both. The addition of silica spheres is used to support the nanoparticles, which avoid the poor catalytic efficiency for the flocculation of nanodimensional materials during the processes of catalytic degradation processes using AgNPs [92]. Activated carbon loaded with AgNPs was suggested to have high adsorption activity (71.4 mg of MB/g of adsorbent) against methylene blue [93]. The fabrication of AgNPs with nanosilica powder showed 99% removal of dyes such as Eosin yellow, Bromophenol blue 2, CR, and BR upon adsorption. The desorption studies applying acetone showed at 86% desorption of dye, suggesting the novel adsorbent reusability [94]. The nanocomposite of Ag/PSNM (silver/poly (styrene-N-isopropylacrylamide-methacrylicacid)) spheres with catalytic degradation of organic dyes showed high potential application for wastewater treatment [80,87]. Choudhary et al. [80] developed and studied biological/green extracts using a silver nanocomposite with naturally occurring montmorillonite (MMT) clay (MMT/Ag nanocomposite). The author investigated the adsorption efficiency and removal of MB dye by applying a batch system. This study revealed that the adsorption of two nanocomposites which were raw MMT and MMT/Ag had the capacity to remove MB (methylene blue) [80].

The green synthesised hybrid nanocomposite (Brassica nigra) of rGO-AgNP showed antimicrobial activity and efficient photocatalytic activity in direct blue 14 (DB-14) dye. It exhibited a high photocatalysis performance in dye removal with sunlight compared to ultraviolet (UV) and could be reused for five times without a significant loss of photocatalysis performance [95]. The ultrasonic synthesised Ag/CTAB/NCC (nanohybrid) without acid hydrolysis had a stronger catalytic property than other catalysts and showed better removal of methyl orange (k = 14.2 × 10^−3^ s^−1^, t = 150 s) and 4-nitophenol (k = 5.4 × 10^−3^ s^−1^, t = 180 s) [88]. The one-dimensional AgNP/WPI-AF (amyloid-based hybrid) materials were fabricated using photochemical/chemical routes. The selective support of the AgNP (silver nanoparticle) amyloid fibril (AF) was derived from WPI (whey protein isolate) for the catalytic reduction/removal of the MB (methylene blue) dye. The material of the nanoparticle-amyloid fibril composite is a better example of the process of catalysis, and it showed better reusability [85]. The fabricated nanocomposite of Ag@MGO-TA/Fe^3+^ showed catalytic reduction performance against organic pollutants and antimicrobial performances, especially disinfection action against bacteria (*E. coli*) [70].

**Figure 5:**
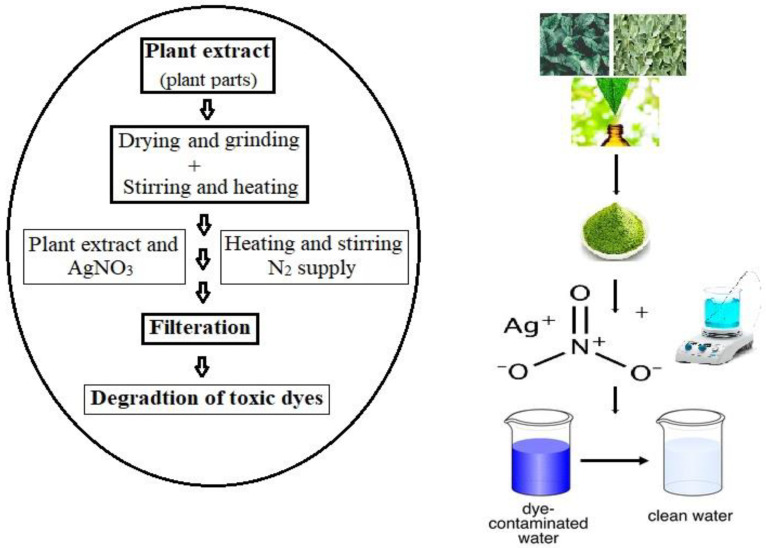
Diagrammatic representation of AgNPs (SNPs) from plant extract and their use as a dye degradation (adapted from [69]).

The preparation of CNF/PEI/AgNP composites was developed from the cellulose nanofiber (CNF) from cross-linked bleached birch kraft pulp with polyethene imine (PEI) and decorated with silver nanoparticles (AgNPs). It exhibited shape memory properties and good mechanical stability under wet conditions, and its decolorization activity was high as 5 × 10^4^ Lm^−2^ h^−1^. This study demonstrated the recyclability and stability of the 3D nanocellulose-based aerogel membrane after a continuous catalytic discoloration process was performed ten times [79,96,97]. In organic compound degradation, semiconductor nanomaterials are widely used as the photocatalyst. During the photodegradation, the nanoparticles were separated from the treated solution. Therefore, to avoid this problem, the author developed a novel cross-linked membrane and achieved fast degradation of 98% for the Ag/rGO nanocomposite and 92% for Ag/rGO/CA/TFC membranes [78]. Figure 5 shows a schematic representation of AgNPs (silver nanoparticles) from a plant extract and their use as dye degradation.

## 5. Silver Nanoparticles in Agriculture

**Figure 6:**
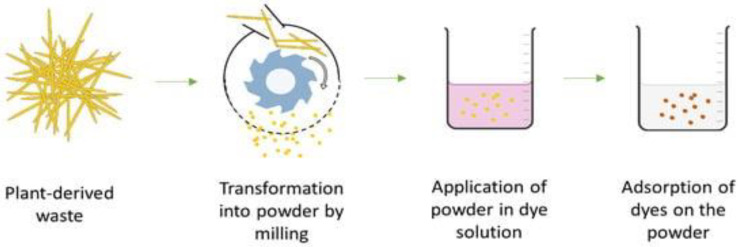
Anionic dye removal by plant-derived agricultural waste mechanical modification (adapted with permission from [100]).

AgNPs may synthesize processes by physical and chemical methods. They contain varying potential features that make them “adverse” combination methods. The making of AgNPs from biological techniques has emerged as an outcome of research for such a technology. The nanoparticle fabrication is completed by a wide range of plant families and microorganisms using the methods of reduction/oxidation processes. Photochemical techniques react with the materials to produce nanoparticles that we require as a solvent medium: harmless/non-toxic eco-friendly stabilising agents. Many researchers have synthesised AgNPs from plant extracts and microorganisms such as bacteria, fungi, algae, etc. [98,99]. Figure 5 shows an illustrative explanation of the green synthesis of AgNPs from plant parts and microorganisms, their characterisation, and their activity. The mechanical modification that is particularly involved in the mechanical-milling operation is shown in Figure 6.

**Figure 7:**
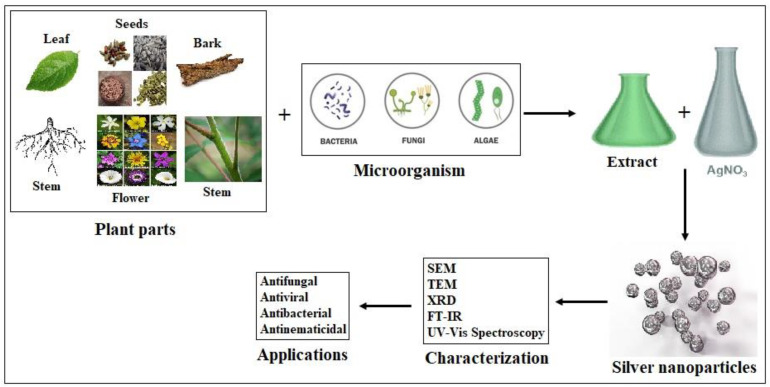
Schematic representation of the green synthesis of AgNPs from plant parts and microorganisms, their characterisation, and their activity [108].

Silver nanoparticles (AgNPs) have been shown to increase plant growth, seed germination, and crop yields. Additionally, they influence the response of the plant growth to positive/negative impacts. The application of AgNPs transforms the bacterial diversity in soil and influences plant growth in that soil. The various concentrations of AgNPs cause changes in the functional bacterial diversity. The combination of microbes and plants with silver nanoparticle interactions is complicated; by arranging the concentration of AgNPs, the plant growth potential can be increased without affecting the environment [101]. In addition, AgNPs significantly enhance the potential of seed germination, index, mean germination time, index of seed vigour, and fresh and dry seedling weights. The colloidal AgNPs contain significant characteristics of stabilised and well-dispersed characteristics showing more adhesive on the surfaces of the bacterial and fungal cells, hence behaving as excellent bactericides and fungicides. They also enhance the control of plant diseases in food crops and fruits that are economically important. Worldwide, bacterial diseases cause a significant loss in crop yields. Silver nanoparticles were found to act against the activity of plant pathogenic bacteria. It explained that silver nanoparticles have a higher antibacterial activity than generic antibiotics [102,103]. These nanoparticles have been tested as pesticides, and they reduce the burden of pests. This often decreases the use of chemical-based fertilisers in conventional agriculture. Silver nanoparticles have better antibacterial activity observed against nosocomial infections, and their combination with cephalosporin antibiotics resulted in an effective treatment for Pseudomonas infections [104]. Figure 7 represents the schematic representation of the green synthesis of AgNPs from microorganisms and plant parts, their activity, and their characterization. The economical use of water sources by the use of treated water for agriculture and other industrial purposes and the utilization of low-cost and innovative environment-friendly effective paths helps to conserve the limited clean water reservoir and is the best way to save the world’s freshwater [105]. Green-synthesized plant-mediated extracted AgNPs have enhanced the properties of catalytic activity, are chemically stable with the ratio of high surface volume, and can be employed for freshwater and agricultural wastewater treatment [106,107].

Silver nanoparticles are an effective antimicrobial agent against plant pathogens, and they control colony formation and pathogenic plant diseases (fungi) (Bipolarissorokiniana and Magnaporthe grisea). They inhibited the fungi growth (Aspergillus parasiticus) and decreased the synthesis of aflatoxin B1, secondary metabolites, and carcinogenic mycotoxins [109]. Citrate-coated AgNPs improved rice production effectively and protected plants against rice pathogens due to antibacterial activity [110]. Furthermore, the AgNPs stabilising fructose showed antimicrobial effects against phytopathogens, such as Dickeyasolani, Erwinia amylovora, Xanthomonas campestris, Clavibactermichiganensis, and Ralstonia solanacearum [111,112,113]. The silver nanocomposite of GO-Ag NPs (Graphene oxide–silver nanoparticles) was used to treat spot disease found in infected leaves and was applied to Fusarium graminearum. These nanocomposites have been found to inhibit spores and fungal hyphae [114]. Silver nanoparticles are effective in increasing agricultural production *and it was affected by the bovine herpes virus’s activity* [115]. The silver cellulose matrix enhances the adherence character of the foliage of the patches, and it can enhance the action of pesticides. Silver nanoparticles have been used for their antimicrobial activity against Alternaria solani (fungus); they inhibit/diminish the pathogenic population of both in vitro and in vivo conditions of early blight disease in tomatoes in a concentration-dependent manner [116]. The silver nanoparticle-functionalised nanocomposite (polyaniline-reduced graphene oxide/Ag-PANI/rGO) developed non-enzymatic electrochemical glucose sensors with effective sensitivity and a rapid response time; this nanocomposite is an efficient electrochemical method for sensing glucose in samples such as milk and juices [117].

## 6. Effect of Textile Dyes on Health and the Environment

The global textile industry consumes the highest volumes of raw water. This is one of the main industries growing proportionally while increasing the demand for worldwide textile products [118]. Spinning, weaving, finishing, washing, bleaching, stabilising, and dyeing are major operations of the textile industries. The unsuitable disposal waste of textile sectors such as dyes is causing severe environmental health problems. The global textile sector produces 7–10 million tons of dyes yearly, and there are communally more than one million types of dyes [119]. According to the usage and utilisation techniques of dyes (direct, reactive, vat, disperse azo, acid, and anthraquinone dyes) by which they are generally classified, all organic dyes, especially azo dye, hold up to around 70% of the market share. The textile effluents contain colour, TDS (total dissolved salts (TDS), COD (chemical oxygen demand), pH, and turbidity, which are the major constituents of dye effluents. The effluent dyes affect the water’s aesthetic value and possess harmful health and environmental threats. They influence normal aquatic life and are carcinogenic for humans. Azo family dyes and anthraquinone dyes, such as Disperse Blue 3, are found to have carcinogenic threats and intense toxic effects. Several vital azo dyes degrade the environment of the intestine into amines, which are known carcinogens [120].

The degradation/removal of dyes from industrial effluents/wastewater poses a major challenge. Adsorption, granulated/powdered activated carbon (physical), coagulation (chemical), and microbial degradation/fungal decolourization (biological) are being applied for the removal of dyes from wastewater in current practises by the industry [121].

Living organisms require risk-free, nonpolluted water to regulate their metabolism and temperature. Anthropogenic activity can cause water contamination that results in terrible environmental problems. The growth of synthetic chemical fabrication and utilisation has contaminated the waterbodies over the years. Most of these waterbodies around industrial zones have been contaminated by the textile industries. The effluent of the dye can damage the whole ecosystem and associated plant life when it is affected by the chemicals synthesized from toxic organic dyes [122].

**Figure 8:**
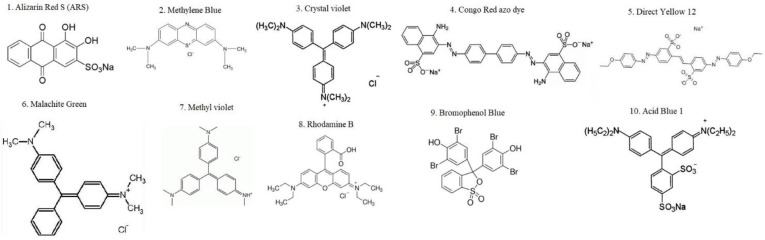
Chemical structures of dyes in textile wastewater.

The wastewater from the textile industry develops large varieties of chemical pollutants and dyes [60]. The complicated chemical structures of a few dyes/pigments are given in Figure 8. The removal of dyes from industries (textile) and wastewater from the dye-making industry has been a significant environmental challenge [123].

## 7. Challenges in Environmental Safety

This section is not mandatory but may be added if there are patents resulting from the work reported in this manuscript.

**Figure 9:**
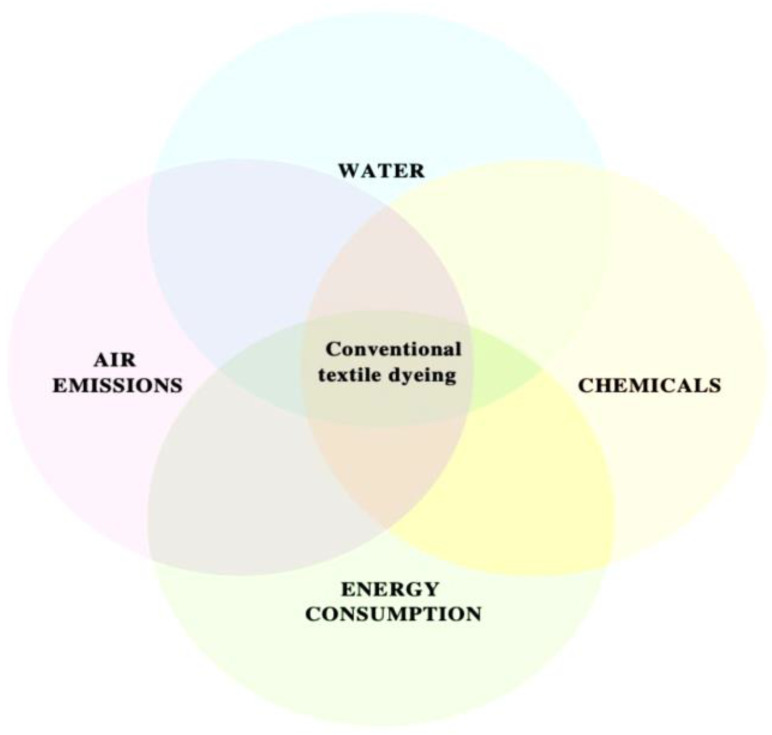
Impact of the dyeing process on environmental systems [128].

The modern world has access to innovative applications that enhance the standard of life. In this way, the textile industrial sector expanded to meet the needs of the population by generating a vast amount of industrial-based goods. Finally, most industrial waste that contributes to environmental contamination has been eliminated. Industrial waste was disposed of on land, producing non-biodegradable waste and non-agricultural processes [124]. In contrast, industrial dye water combined with water sources such as ponds, lakes, rivers, and the ocean to pollute aquatic life. This dye waste exacerbates the ecological dilemma of many diseases affecting land- and water-dwelling organisms. Dangerous chemicals must be filtered out of industrial wastewater before it is discharged. Controlling textile dye *wastes* was critical for reducing wastewater pollution and to maintain the ecological system of earthly life [125,126]. The dye from textile industry is part of the water utilized to colour the prepared cloths. In this process, synthetic dye is mostly toxic chemical elements that are added to apply the colour to cloths. After the colouring process is completed, the dye wastewater creates a pollutant of the ecosystem [127]. The wastewater produced by the dye results in the environmental system shown in Figure 9.

The textile dyeing method is used to enhance the aesthetic appeal of fibres, yarn, and finished products. Plants, seeds, roots, leaves, cellulose, animals, minerals, etc. are utilised to extract natural pigments [129,130]. This form of dyes has minimal impact on the eco-system since this organic ingredient is readily biodegradable by bacteria. However, the synthetic dyes used in the colouring process are not biodegradable. This textile dyeing process has an influence on the environment due to its chemical, water, and air emissions and energy consumption [131,132]. Synthetic dye components are composed of additional chemicals used to combine large amounts of water [132,133]. Presently, 20% of textile dyeing wastewater is discharged by globalisation-related companies. Most synthetic colours are produced from large quantities of chemicals, acids, salts, and peroxides [134]. This strong chemicals combine directly with water to affect ecological systems. This dyeing process pollutes the water’s BOD and COD levels, resulting in the demise of aquatic life. It was more hazardous for acidic and flammable textile chemical acids to react, *and they damage* both aquatic and terrestrial life systems. The cloth dyeing procedure requires heating and cooling, which consume more electricity. This electricity was generated by burning coal and other fossil fuels, contributing to air pollution [135]. Constant production of carbon dioxide by the electricity generation process poses a major threat to the ozone layer. All the environmental contamination caused by the textile dyeing process was decreased by the water treatment technique.

## 8. Conclusions and Perspectives

This review paper summarises recent literature on the importance of the AgNP-based composites for adsorbing or degrading (catalytic/photocatalytic degradation) the textile dye and for the challenges and applications in environmental protection in agriculture. The literature review clearly demonstrated the degradation/removal of textile dyes from wastewater using nanoparticles incorporated with membranes to degrade toxic compounds. The treatment efficiency showed that AgNPs were highly superior to their widespread, as demonstrated by several experimental results. The experimental results in the literature explained that biological extracts of AgNPs from plant materials make better changes and help to protect the environment from harmful damage caused by the extreme utilisation of chemicals. The most significant advantage is the recovery of silver nanocomposites and the reusability of the material for the next cycle. The main advantage of AgNPs for the removal of microbes is through the antimicrobial activity of silver particles, the degradation of organic chemicals/pollutants/dyes by adsorption, and catalytic/photocatalytic activity for treating polluted/wastewater. Although nanoparticles are apparently shown to provide numerous potential advantages for water treatment/purification, there may also be numerous obstacles before they can be executed for extensive applications. Thus, several investigations are required to control these obstacles by planning suitable conversions of silver NPs to fully grasp their possibilities.

## Figures and Tables

**Table 1:** Nanoparticles for textile dye removal.

No	Nanomaterial Type	Type of Process	Nanoparticle Material	Textile Dyes	Removal Efficiency	References
1	Powder	Photocatalytic and microwave-assisted degradation method	ZnO/poly (1-naphthylamine) nanohybrids	Alizarin red	85%	[38]
2	Powder	Catalytic degradation method	Pd	Azo dyes	93 and 91%	[40]
3	Powder	Catalytic degradation method	Cu	Methyl orange	Less than 80%	[41]
4	Powder	Photocatalytic degradation method	Fe_2_O_3_	Acid blue	87%	[42]
5	Decorated	Enzymatic reaction	Fe_3_O_4_	Acid fuchsin	Up to 80%	[43]
6	Powder	Photocatalytic degradation method	Ag	Methyl orange and Coomassie brilliant blue	60%; 70%	[44]
7	Powder	Biogenic method	Biogenic Pd	Acid blue 1 and red, methyl orange and reactive black 5	Less than 95%	[37]
8	Powder	Photocatalytic degradation method	Ag–ZnO/GO	Methylene blue	85%	[45]
9	Powder	Photocatalytic degradation method	ZnO/CuO	Methylene blue	93%	[46]
10	Powder	Adsorption	Fe_3_O_4_	Optilan blue	50 mg/L with 0.6 g/L	[47]
11	Powder	Desalination	GO-PEG-NB	Ternary dyes	99%	[48]
12	Powder	Adsorption–photocatalysis	Ze-nanZnO; nanZnO	Tartrazine	87 and 81%	[49]
13	Film	Adsorption	CS/MgO	Reactive blue (RB) 19	77.62%	[50]
14	Powder	Adsorption	CS–ZnO	Malachite green (MG)	98.5%	[51]
15	Powder	Photocatalytic degradation method	TiO_2_ + MC (micro cellulose)	Methylene blue, methyl violet and acid violet	99%	[52]
16	Powder	Photo degradation method	CS/ZnO	Methylene blue	CS: 86.7%; MB: 81%	[53]
17	Powder	Photocatalytic degradation method	ZnO/AC	Methylene blue	92.2%	[54]
18	Powder	Adsorption	CHT-GLA/ZnO/Fe_3_O_4_	Brilliant Blue R	176.6 mg/g at 60 °C	[39]
19	Ni@FP	Coated on Cellulose filter paper	Dyeing wastewater	Methylene orange	93.4%	[55]
20	Dry powdered gel	Photocatalytic degradation	LaFeO_3_- RGO–NiO	Congo red	96.5%	[56]
21	Powder	Photocatalytic degradation method	Ag–ZnO	Methylene blue, methyl orange and rhodamine B dyes	98.5%	[57]
22	Powder	Photocatalytic degradation method	ZnO	Methylene blue	90%	[58]
23	Powder	Photocatalytic method	ZnO	Alizarin red S (AZ) and methylene blue (MB) dyes	99.9 and 96.8%	[59]
24	Powder	Photocatalytic degradation method	CuO	Methylene blue (MB)	93%	[60]

**Table 2:** Sliver nanoparticles for dye removal.

No	AgNPs-Composites	AgNPs-Composites Synthesis Method	Type of Pollutant	Name of the Pollutant	Treatment Efficiency	References
1	AgNPs capped 2-hydroxypropyl β-cyclodextrin/alginate nanocomposite	Leave extract from Jasminum subtriplinerve	Organic pollutant and dyes	4-NP, MO, rhodamineB	Kinetic (pseudo-first order) rate 1.51 × 10^−3^ s^−1^ to 2.23 × 10^−3^ s^−1^	[71]
812	Silver nanoparticles (AgNPs)	Leave extract from Thymbra spicata	Organic pollutant and dyes	4-NP, MO andrhodamine B	Catalytic activity loss	[72]
3	FeO/AgNPs (Fe–Ag core-shell nanoparticles)	Pomegranate fruit peel extract	Dyes	Aniline blue dye	90%; 0.25 mg mL^−1^	[73]
4	Fe_3_O_4_/PPy-MAA/Ag	Polymer matrix	Organic pollutant and dyes	4-NP and MB, MO	42.5 wt% (20 min)	[74]
5	Silver-doped Mg_4_Ta_2_O_9_ nanoparticles	Irradiation of UV lamp	Dyes herbicide	rhodamine B, methyl orange, atrazine	2.0 wt%	[75]
6	Cellulose polymer paper in silver nanoparticles	Leave extract from Durantaerecta	Organic pollutant	4-NP, 2-NP (2-nitrophenol), (2-Nitroaniline) 2-NA, TNP	6–12 min, Stable catalyst for five cycles. 95–99%	[76]
7	TiO_2_/CNTs/AgNPs/Surfactant (C10) nanocomposite	Trisodium citrate solution	Dye	Methylene blue (MB)	Degraded in 180 min; 0.5 gL^−1^, 100%	[77]
8	CAg-NPs	Citrus paradisi	Dye	Congo red (CR), MB, malachite green (MG), rhodamine B (RhB) and 4-NP	MB: 93.29; MG: 83.73; 4-NP: 88.9; RhB: 60.53	[78]
9	CNF/PEI/Ag NPs composite	Bleached birch kraft pulp	Organic dye	MB	96% after 4 min	[79]
10	rGO-AgNP (graphene oxide silver nanoparticle hybrid nanocomposite)	Brassica nigra aqueous extract	Dye	Direct blue-14 (DB-14)	95.41%	[80]
11	GO−ZnO−Ag	Simple one-pot method	Organic dye	MB	100%, 40 min	[69]
12	AgNPs/holocellulosenanofibrils (AgNPs/HCNF)	Microwave-assisted	Dye	MB	94–98%, catalytic activity with five cycles	[81]
13	AgNPs/ZIF-8 composite	NaBH4 and trisodium citrate solution	Dyes	MB and CR	MB: 97.25%; CR: 100% pH ≥ 7	[82]
14	AgNPs impregnated sub-micrometercrystalline jute cellulose (SCJC) particles	Extract of leaves of M. erythrophylla	Dyes	CR and MB	100%, 14 min with 0.005 mg/mL	[83]
15	AgNPs	Extract of leaves from Portulacaoleracea (PNL)	Textile dyes	Reactive green 19A, R blue 59, R red 120, R red 141, and R red 2	180 min, 50; 35% fourth and fifth cycles	[84]
16	Ag@MGO-TA/Fe^3+^ nanocomposite	Graphite flakes	Organic pollutants	Methylene blue	0.05 mg/mL	[85]
17	CH-AgNPs	Trisodium citrate solution	Dye	Orange and blue dyes	97.4 and 100%	[86]
18	MMT/Ag nanocomposite	Montmorillonite (MMT) clay and AgNPs	Dye	Methylene blue	99.90% for 25 ppm; 96.50% for 50 ppm; 89% for 100 ppm and 81.14% for 200 ppm	[87]
19	Ag/CTAB/NCCnanohybride	Microcrystalline cellulose	Dye	Methyl orange, 4-nitrophenol	14.2 × 10^−3^ (s^−1^); 5.4 × 10^−3^ (s^−1^)	[88]
20	Ag/rGO nanocomposite and Ag/rGO/CA/TFC membranes	-	Organic compounds	Methylene blue	98%; 92%	[78]
21	FBN-GO-Ag	-	Wastewater	Reactive black 5 and reactive red 120	88.9 and 77.7%	[89]
22	BaTiO_3_/AgNPs	BaTiO_3_	Dye	Methylene blue and ciprofloxacin	72 and 98%	[90]
23	AgNP/WPI-AF	Whey protein isolate	Dye	Methylene blue	-	[85]
24	AgNPs decorated on nanostructured porous silicon	Peumo extract	Organic dyes	Methylene blue	Degradation rate 8.6/min	[91]

## Data Availability

Not applicable.
